# LONG-TERM OUTCOMES OF MODERATE TO SEVERE DIFFUSE AXONAL TRAUMATIC BRAIN INJURY: A PROSPECTIVE STUDY

**DOI:** 10.2340/jrm-cc.v8.42299

**Published:** 2025-04-03

**Authors:** Marianne LANNSJÖ, Jörgen BORG, Anders LEWÉN, Charlotta VON SETH, Per ENBLAD, Sami ABU HAMDEH

**Affiliations:** 1 Department of Medical Sciences; Section of Rehabilitation Medicine, Uppsala University, Uppsala, Sweden; 2 Karolinska Institutet, Department of Clinical Sciences, Danderyd Hospital, Division Rehabilitation Medicine, Stockholm, Sweden; 3 Department of Medical Sciences; Section of Neurosurgery, Uppsala University, Uppsala, Sweden; 4 Department of Medical Sciences, Uppsala University, Uppsala, Sweden

**Keywords:** traumatic brain injury, diffuse axonal injury, long-term outcome, GOSE

## Abstract

**Introduction:**

Traumatic brain injury (TBI) with diffuse axonal injury (DAI) necessitates significant medical and rehabilitation interventions. The late long-term outcome is variable with potential for neurodegenerative development and deterioration. This study evaluates the late long-term outcomes of moderate to severe TBI with DAI.

**Methods:**

Patients aged 16–65 with moderate to severe TBI and DAI were included. From 2006 to 2018, 30 patients (mean age 34; 21 males, 9 females) were enrolled. Outcomes were assessed using the Glasgow Outcome Scale Extended (GOSE) at 6 months and ≥ 1-year post-injury.

**Results:**

At 6 months, 10 patients had a favourable outcome (GOSE 6–8), increasing to 12 at ≥ 1-year post-injury. Patients with unfavourable outcomes were older (mean 40) than those with favourable outcomes (mean 24, *p* < 0.001). DAI stage correlated with outcomes (*p* = 0.003). GOSE remained unchanged in 15 patients, improved in 9 and deteriorated in 6 between the 6 months and the ≥ 1-year follow-up.

**Discussion:**

Approximately one-third of TBI patients with DAI achieved favourable long-term outcomes, and the outcome changed in half of the patients between 6 months and ≥ 1 year follow-up. Age and DAI stage were significant predictors of outcome. Further studies are required to enhance prognostic accuracy and explore rehabilitation’s impact.

## LAY ABSTRACT

Traumatic brain injury (TBI) with widespread damage to the brain’s nerve fibres, a condition called diffuse axonal injury (DAI), necessitates intense medical and rehabilitative interventions. The long-term outcome is variable, and some patients may deteriorate over time. This study investigated the long-term outcomes of patients with moderate and severe TBI with DAI. Thirty patients, aged 16–65 years, who had suffered moderate to severe TBI with DAI were followed, and the recovery was evaluated at 6 months and > 1 years post-injury. After 6 months, 10 patients had a good recovery, which increased to 12 patients after > 1 year. Patients with poor outcomes were older (average age 40) than those with good outcomes (average age 24), and the severity of DAI was associated with recovery. We conclude that one-third of DAI patients achieved a favourable outcome. Age and DAI severity were factors associated with recovery.

Traumatic brain injury (TBI) is a common condition leading to hospitalization and need of rehabilitation services. TBI can be classified into mild, moderate and severe injury, based on the patient’s clinical presentation, and brain imaging data may add prognostic information (1). The incidence of TBI across all injury severities has been estimated to 118–546/100,000 individuals per year in Europe (2). For severe injuries only, the incidence was found to be 4.1–17/100,000 per year (2). Another review of TBI incidence in Europe reported a range of 11–47/100,000 per year for moderate to severe TBI (3).

Diffuse axonal injury (DAI) in TBI is an important mechanism influencing the degree of injury and long-term outcomes. DAI encompasses a spectrum of abnormalities from primary mechanical breaking of axon cytoskeleton, to axonal transport interruption, swelling and proteolysis, through secondary physiological changes (4). DAI is commonly classified into 3 stages (5) of increasing severity (*i*. microscopic-only evidence of axonal damage in cerebral hemispheres, *ii*. the addition of focal lesion in corpus callosum, *iii*. the addition of focal lesion in the brain stem). To provide a more differentiated description of magnetic resonance imaging (MRI) findings in DAI and their relation to outcome, Abu Hamdeh et al. (6) have proposed an extended MRI classification system based on 4 stages: (*i*) Hemispheric lesions, (*ii*) Corpus callosum lesions, (*iii*) Superficial brainstem lesions and (*iv*) Substantia nigra or mesencephalic tegmentum lesions.

Whilst DAI has been associated with the poorest outcomes post-TBI (6–8), several studies have indicated that a significant proportion may have a rather favourable outcome 1 year after injury (9–11). Brainstem injury and higher DAI stage have been linked to poorer outcomes (9–12).

The late long-term outcome following TBI with DAI is of importance due to a substantial risk for eurodegeneration observed (4, 13, 14). Lesion localization and DAI grade may predict grade of atrophy, and neuropsychological testing has revealed a correlation between atrophy grade and cognitive outcomes (14). The patterns of neurodegeneration observed post-TBI are distinct, predominantly located in white matter and differ from Alzheimer’s disease and normal ageing (15).

There are few studies on adults examining outcomes > 1 year after TBI with DAI. In a study with 3–6 years follow-up (mean 5 years) (16), DAI grade did not predict outcomes using the Glasgow outcome scale extended (GCS). Another study (17) with a median follow-up of 54 months (range 14–100) reported favourable GOSE (defined as GCS 6–8) in 39% of those with DAI III and 47% in DAI II. More studies are warranted to elucidate the long-term outcomes after TBI with DAI.

“What is the prognosis” is a common question from patients and kindred after TBI. The question must be answered responsibly. Even if there is evidence supporting favourable outcome for a substantial proportion of patients with TBI and DAI, there is a potential risk for neurodegenerative development. Furthermore, there are persisting uncertainties regarding the prognostic value of DAI lesions for the late long-term outcome. This study therefore aimed to evaluate the late long-term outcome in comparison with the outcome at 6-months post-injury, focusing on moderate or severe TBI with DAI, using a 4-level DAI-grading scale (6).

## METHODS

The Regional Research Ethics Committee granted permission for all included studies. A written-informed consent was obtained from the TBI patient’s closest relative, and all research was conducted in accordance with the ethical standards given in the Helsinki Declaration of 1975, as revised in 2008.

### Patients

Inclusion criteria for this study were as follows: aged 16–65, documented head injury, a Glasgow coma scale (GCS) score of 3–12 and a diagnosis of DAI in the acute phase by MRI or by a combination of computed tomography (CT) and clinical assessment. Patients with previous significant TBI, neurological or psychiatric disease, substance abuse or other coincidental conditions of importance were excluded from the study. Between the years 2006 and 2018, a total of 69 individuals were enrolled in the study. All patients were admitted for neurointensive care at the University Hospital in Uppsala, Sweden.

An initial CT was performed on admission and scored according to the Marshall classification (18). Suspected DAI on the initial CT was subsequently confirmed with MRI when the patient was medically stable and possible to transfer to the radiology department. MRI was performed with a 1.5T scanner (Siemens Avanto, Siemens Healthineers, Erlangen, Germany). Imaging included a T2*-weighted gradient echo (T2*GRE) sequence (repetition time [TR]: 500 ms, echo time [TE]: 14 ms, flip angle [FA]: 30 degrees, acquisition voxel: 0.9 × 0.9 × 3 mm), a diffusion-weighted (DW) sequence (Spin-echo Echo-planar Imaging; (SE-EPI), b-value 1000 s/mm^2^, TR: 4700 ms, TE: 89 ms, acquisition voxel: 1.2 × 1.2 × 5 mm) and a susceptibility-weighted (SWI) sequence (TR: 49 ms, TE: 40 ms, FA: 15 degrees, acquisition voxel: 0.9 × 0.9 × 1.5 mm). Haemorrhagic lesions were assessed on T2*GRE and/or SWI sequences depending on availability. DAI-associated lesions were defined as hypointense/decreased signals on T2*GRE and SWI sequences, and/or high signal intensity on DW images in white matter structures not extending to the cortex. Adams’ anatomical histopathological grading system was applied (Grade I – Lesions in cerebral hemispheres only, Grade II – Lesions in corpus callosum and Grade III – Brainstem lesions) (2). In addition, DAI staging according to previously published work by our group was performed using all sequences combined (Stage I – Lesions in cerebral hemispheres only, Stage II – Lesions in corpus callosum, Stage III – Lesions in the brainstem excluding the substantia nigra and mesencephalic tegmentum (SN-T) and Stage IV – Lesions in SN-T).

### Outcome

A first follow-up was performed at around 6 months according to the GOSE. Patients were assessed by a neurointensive care specialist nurse, trained to perform the structured interview and blinded to all clinical data. A standardized questionnaire was used, based on the structured interviews by Wilson et al. (19), to be filled by patients or closest relative. When supplementary information was needed, patients or relatives were contacted by telephone. A second follow-up interview at least 1 year after injury was made by telephone or by a visit. The interview, which was performed by 2 of the authors, both rehabilitative medicine physicians (ML and CS), consisted of a comprehensive assessment which included the estimation of GOSE again and the length of post-traumatic amnesia (Rivermead post-amnesia protocol; post-traumatic amnesia [PTA]) (20).

GOSE is a widely used instrument for assessing global outcome after TBI and has been shown valid and reliable (19, 21–24). It consists of an ordinal scale with 8 steps, where 1 = dead, 2 = vegetative state, 3 = lower severe disability, 4 = upper severe disability, 5 = lower moderate disability, 6 = upper moderate disability, 7 = lower good recovery and 8 = upper good recovery (25). Rivermead PTA protocol estimates time length of PTA by clinical questioning of the patient. Four grades of PTA are defined by establishing how long after injury (in hours/days/weeks) the patient regains continuous day to day memory: mild = less than 1 h, moderate = 1–24 h, severe = 1–7 days and very severe = more than 7 days (20).

### Statistics

All statistical analyses were conducted in SPSS version 27 (IBM Corp., Armonk, New York, USA). Χ^2^ tests, Mann-Whitney *U* tests and Kruskal-Wallis tests were performed to test statistical differences between groups. A *p*<0.05 was considered statistically significant.

## RESULTS

From a total of 69 individuals, 8 died prior to follow-up, 2 lived abroad and were therefore inaccessible for contact, 3 patients declined to participate, whilst 21 did not respond. Non-responders did not differ significantly from responders in age (*p* = 0.38), gender (*p* = 0.34) or TBI severity (*p* = 1.0). Of the remaining 35, GOSE classification at the 6-month follow-up was unavailable for 5 individuals. Thus, 30 patients (21 males, 9 females, mean age 34, range 16–61 years) with a follow-up data at 6 months and > 1 year post-injury were included in the study. Demographic data are provided in Table I.

**Table I:** Demographic and clinical data of traumatic brain injury patients.

Variable	No
Gender	
Men	21
Women	9
Glasgow coma scale	
3–8	27
9–12	3
Cause of traumatic brain injury	
Traffic, inside car	19
Pedestrian	2
Bicycle	3
Sports	5
Fall	1
Diffuse axonal injury staging by Abu Hamdeh^6^	
I	2
II	6
III	6
IV	14
Missing	2

At admission to hospital, 27 patients were classified as severe TBI (GCS 3–8), whereas 3 patients had suffered a moderate TBI (GCS 9–12). Long-term follow-up was performed at a mean of 5 (range 1–14) years post-injury, by telephone interview in 12 patients and an in-person visits in 18 patients. MRI was performed after a mean of 5 (range 1–141) days post-injury. In 2 patients, an MRI could not be performed, but the diagnosis of DAI could be made based on a combination of CT findings and clinical criteria. Twenty patients were classified as DAI grade III according to Adams classification. Amongst them, 14 were classified as DAI stage IV according to classification proposed by Abu Hamdeh et al. (Table I). Twenty-four participants exhibited PTA lasting more than 7 days during the follow-up, whereas 4 patients classified as GOSE 3 were unable to provide a response. In 2 patients, PTA data were missing. Three patients developed post-traumatic hydrocephalus and underwent ventriculoperitoneal shunt (Codman-Medos-Hakim) surgery at 1–7 months post-injury.

At 6 months, 10 patients achieved a favourable outcome, defined as a GOSE score of 6–8 (Table II). At the second follow-up, 3 more patients achieved a favourable outcome, and 1 patient deteriorated to an unfavourable outcome (Table II). Mean age at the time for injury for those achieving an unfavourable outcome (*n* = 18) at the second follow-up was 40 years (range 21–61), and for patients achieving a favourable outcome, the mean age was 24 years (range 16–52, Mann Whitney U test, *p <* 0.001). Seven out of eight patients classified as DAI stage I-II, 2/6 patients classified as DAI stage III and 2/14 patients classified as DAI stage IV achieved a favourable outcome at the second follow-up (Table II). There was a significant association between the DAI stage and outcome measured by GOSE at the second follow-up (3-group Chi square test, *p* = 0.003 (DAI I-II vs DAI III, *p* = 0.036; DAI I-II vs DAI IV, *p* = 0.00078; DAI III vs DAI IV, *p* = 0.33)), but there was no significant difference between the DAI stages III and IV (post-hoc test, *p* = 0.33).

**Table II:** Glasgow outcome scale extended at 6 month and second follow-up ≥ 1 year including acute data for age and diffuse axonal injury (DAI) stage.

GOSE 6 months	GOSE second follow-up	Age at injury (mean)	DAI stage
3	3	21	IV
3	3	24	IV
3	3	34	IV
3^†^	3	35	IV
3^†^	3	48	IV
3	3	60	IV
5	5	22	III
5	5	36	III
5	5	38	III
5	5	47	-
5	5	52	IV
6	6	18	I
7	7	30	II
8	8	16	II
8	8	18	III
3^†^	4	28	IV
3	4	58	IV
3	4	61	IV
4	5	28	IV
4*	6	16	I
5*	7	20	IV
5*	8	52	III
6	7	19	-
6	8	25	II
5	4	36	III
5	4	58	IV
7	6	19	II
7*	4	42	II
8	7	17	II
8	7	35	IV

GOSE: Glasgow Outcome Scale Extended.

Green colour for improvement, red for deterioration and grey for unchanged.

* Change of category favourable/unfavourable,

† Ventriculoperitoneal shunt surgery for post-traumatic hydrocephalus.

**Fig. 1:**
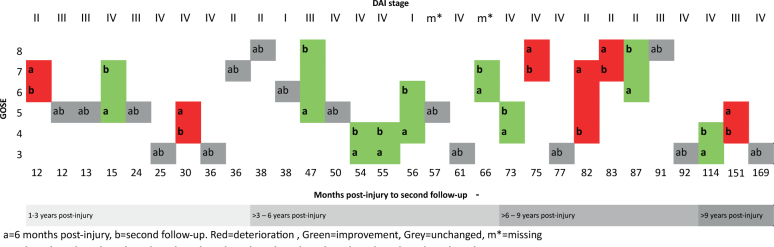
Glasgow outcome scale extended at 6 months and at second follow-up ≥ 1 year for each patient, related to time at follow-up in months. GOSE: Glasgow coma scale extended; DAI: Diffuse axonal injury; a = follow-up at 6 months post-injury, b = second follow-up, red = deterioration, green = improvement, grey = unchanged.

The GOSE score was unchanged in 15 patients, improved in 9 and deteriorated in 6 patients between the 6 months and the second follow-up. Ten patients changed GOSE category (severe disability, moderate disability or good recovery), and 4 patients changed categories between favourable and unfavourable outcome, with 3 improving and 1 deteriorating (Fig. 1). There were no significant association between changes in GOSE between the 2 follow-ups and age or DAI stage (Kruskall-Wallis test *p* = 0.98 and *p* = 0.86, respectively).

## DISCUSSION

In this study, we have analysed the late long-term outcomes of TBI patients with DAI. In the cohort, about 1/3 of patients achieved a favourable outcome, defined as GOSE 6–8. The outcome measured with GOSE changed in half of the patients between the 6 months and the second follow-up after ≥ 1 year, improving in 9/30 and deteriorating in 6/30. Age at injury and DAI grade were associated with outcome.

The GOSE is frequently utilized in outcome assessment, but the methods for dichotomizing outcomes as favourable or unfavourable vary between studies. This variation complicates the comparison of study results concerning outcomes. For instance, some studies classify GOSE scores of 7–8 as indicative of a good outcome (26, 27), whilst others use different dichotomizations such as GOSE 1–5 versus GOSE 6–8 (12) or GOSE 5–8 as favourable (28). In the Track-TBI studies (29), a GOSE score of 4–8 is considered favourable, meaning that any state except very severe disability, a non-responsive state, or death is favourable. Despite the frequent use of GOSE in outcome assessment, reliability issues persist (30), affecting the evaluation of results. The decision to classify GOSE scores of 6–8 as favourable in this study is based on the criteria that individuals with these scores possess autonomy at home and in society and can work without any adaptations other than a reduction in working hours. In this study, GOSE assessments were conducted by a limited number of experienced individuals using the GOSE interview method, enhancing the robustness of the results.

The long-term outcome of TBI with DAI is of particular interest due to findings, indicating a risk of neurodegeneration (14). A study by Forslund et al. (31) followed patients with moderate to severe TBI across various injury types at intervals of 1-, 2-, 5-, and 10-years post-injury. This study revealed that 56% showed no change in their GOSE scores, 37% deteriorated and only 7% improved. This is consistent with the present study in terms of the proportion of patients with unchanged GOSE scores (15 out of 30), but not with respect to the proportion who improved (9 out of 30). In the current study, 6 out of 30 patients deteriorated, which is lower than the deterioration rate reported by Forslund et al. (31). In contrast, a study from northern Sweden (32) involving patients with severe TBI followed-up at 3 months, 1 year and 7 years post-injury found that disability and cognitive function seemed to improve over time but remained relatively stable from 1 to 7 years. This study did not address DAI. However, a study by Humble et al. (16) focusing on DAI reported that its occurrence was associated with lower Functional Independence Measure (FIM) scores at discharge but not with GOSE scores or quality of life after 5 years. A significant challenge in analysing neurodegeneration in long-term follow-up studies is that GOSE does not explicitly reflect cognitive deterioration and atrophy. In the current study, more patients improved than those deteriorating at the second follow-up > 1 years post-injury, and consequently, it does not support the theory of neurodegeneration following severe TBI with DAI, albeit a small number of participants.

Several factors significantly influence outcomes following TBI and may explain differences between studies, including age, cognitive reserve and the quality of the rehabilitation process. Age is a well-documented determinant of outcome (33). For instance, in a study by Skandsen et al. (27) on moderate to severe TBI with DAI, no association was found between MRI findings and outcomes as measured by GOSE scores. However, amongst patients with moderate TBI, older age was associated with an unfavourable outcome. Abu Hamdeh et al. (6) identified age over 30 years as a strong independent prognostic factor for poor outcome. Similarly, in the present study, age at the time of injury significantly influenced outcomes. The mean age of individuals with an unfavourable outcome was 40 compared to a mean age of 24 for those with a favourable outcome at the second follow-up. However, we did not detect any association between age and changes in GOSE at the second follow-up.

Pre-injury factors such as resilience and cognitive reserve are of importance in predicting long-term outcomes after TBI. Rassovsky et al. (34) found that pre-injury intellectual function predicted work performance, cognitive status, and social and daily functioning 14 years post-TBI. Similarly, Mathias and Wheaton (35) indicated that higher education and pre-morbid IQ are associated with better outcomes after TBI. Sima et al. (36) demonstrated that resilience, defined as positive adaptation and recovery, after trauma, is a significant predictor of long-term outcomes following TBI.

The importance of timely and qualitative rehabilitation has not been extensively studied. However, Godbolt et al. (37) found that delays in rehabilitation admission negatively impacted outcomes in severe TBI. Similarly, Sörbo et al. (38) demonstrated that delayed rehabilitation led to worse outcomes compared to rehabilitation soon after the injury. Although the present study did not include aspects of rehabilitation and cognitive reserve, these factors remain important in a clinical context.

### Limitations

This study is limited by the small sample size and the differences in the time course of the follow-ups. Initially, 69 patients were enrolled in this study. However, for the long-term outcome assessment, 10 patients were unavailable, 3 patients declined to participate and 21 patients did not respond despite multiple contact attempts. We did not observe any significant demographical differences or differences in injury severity between responders and non-responders in the study. Nevertheless, we cannot exclude a selection bias due to the high dropout rate. In addition, we do not have specific information regarding rehabilitative intervention. Nonetheless, neurorehabilitation is well established in our region, and we can be reasonably confident that the patients received adequate rehabilitation after discharge from the neurosurgical clinic.

### Conclusions

We conclude that approximately one-third of TBI patients with DAI achieve favourable long-term outcomes, with half experiencing changes in the outcome between the 6 months and the second follow-up after ≥ 1 year. Age and DAI stage were significant predictors of outcome. Further studies are required to enhance prognostic accuracy and explore rehabilitation’s impact.
